# Investigation of Hydrophysical Properties and Corrosion Resistance of Modified Self-Compacting Concretes

**DOI:** 10.3390/ma17112605

**Published:** 2024-05-28

**Authors:** Adlet M. Zhagifarov, Daniyar A. Akhmetov, Dossym K. Suleyev, Zhanar O. Zhumadilova, Meiram M. Begentayev, Yuryi V. Pukharenko

**Affiliations:** 1 Institute of Architecture and Civil Engineering, Satbayev University, Almaty 050013, Kazakhstan; 2 Department of Civil Engineering, Saint Petersburg State University of Architecture and Civil Engineering, SPbGASU, 4, 2nd Krasnoarmeiskaya Str., St. Petersburg 190005, Russia

**Keywords:** self-compacting concrete, frost resistance, water resistance, corrosion resistance, complex modifier

## Abstract

Improvement of hydrophysical properties and corrosion resistance of self-compacting concrete to the effects of alternate freezing–thawing and aggressive soils of Southern and Central Kazakhstan is of interest to a wide range of researchers from the side of practical application of the obtained results in construction practice. It is proposed to form a spatially reinforced fine crystalline structure of a cement matrix with the maximum dense packing by using a complex modifier (hyperplasticizer + polymer + microsilica + fibro fibers) in the composition of self-compacting concretes (SCCs). The introduction of the calculated amount of the above additives increases the operational reliability of the current SCC compositions, increasing the water resistance to W16, frost resistance to F = 500, increasing the compressive strength by 20%, and reducing the mass loss of samples during corrosion leaching to 50%. It has been experimentally established that the proposed addition of the complex modifier (hyperplasticizer + polymer + microsilica + fibro fibers) to the SCC composition allows obtaining self-compacting concrete of high quality with improved performance characteristics (compressive strength, water resistance, frost resistance, and corrosion resistance). Studies have shown that the complex modifier-modified SCC compositions have a high degree of resistance in aggressive environments and leaching corrosion. Based on the results of the conducted tests, it is possible to recommend the obtained SCC compositions for the production of building products working in the zone of alternating freezing–thawing and aggressive soils.

## 1. Introduction

A promising direction of construction material science is the improvement of technology and study of the operational properties of self-compacting concrete (SCC)—a type of concrete that is increasingly actively used in the construction industry of the Republic of Kazakhstan [1].

The undoubted advantages of SCC are high mobility, ability to compact under the action of gravity, to be placed in densely reinforced structures, and the possibility to eliminate the process of vibration paving of concrete, which improves the quality of concrete structures and provides certain advantages to the construction process. Recently, more attention has been paid in the construction industry to the development of SCCs with improved physical–technical, performance, and durability properties [2]. The composition of SCC differs from that of conventional heavy concrete. In the composition of SCC, there is an increased content of sand and binder (cement and fillers), as well as, properly selected in a quantitative composition, superplasticizers based on polycarboxlates [3]. SCC with a properly selected composition is more homogeneous, and its performance is more similar than that of conventional heavy concrete [4]. At the same time, the requirements for SCCs for the manufacture of building products and structures are different from those for conventional concrete [5]. Such special requirements are primarily due to the specifics of the production, transport, and laying of the SCC. In the process of the operation, the structures from SCC in the southern and central regions of Kazakhstan experience a constant impact of a whole complex of aggressive factors: the impact of groundwater, cavitation’s effect of water flows (on hydromeliorative structures), and frequent temperature fluctuations of ambient air passing through the 0 °C zone. The above-mentioned constantly acting aggressive influences reduce the reliability of structures and gradually destroy structures [6].

This progressively increases the number of defects, occurrence of sinks, and weakening of interstitial partitions and the cement–filler contact zone, which, in turn, leads to a significant loss of strength characteristics of the structure [7]. The relevance of the study lies in the fact that there are very few studies devoted to the study of frost resistance and analysis of hydrophysical properties of self-compacting concretes, as these works mainly consider the study of the strength and deformative properties of SCC with the reflection of the solution of one or two specific problems, for example, solving the problem of crack resistance and shrinkage of concrete [8].

It is possible to increase the durability and improve the operational reliability of structures made from SCC by applying special protective cladding (monolithic and prefabricated), which reduces water filtration through the working surfaces. The use of protective cladding keeps filtration at 15–20%. However, the integrity of the thin protective coatings (0.2 mm thick) is compromised by even minor mechanical impacts, which is a major problem in their use. Scientists are searching for effective materials, for example, polymer compositions capable of resisting both mechanical effects and aggressive environmental influences. Unfortunately, often-proposed polymer compositions involve the use of expensive chemical components, which hinders their widespread use [1]. It should be noted that important measures for the further development and improvement of construction under the influence of an aggressive environment are the improvement of work quality and optimization of the construction time, as well as rational use of the properties of applied concretes and improvement of their quality [9].

Concretes with Portland cement clinker-based binders are still the basis of civil engineering. In modern construction, predominantly modified concretes are used, which make it possible to ensure a given level of quality [10]. Even a small amount of modifying components can dramatically change the process of structure formation and produce durable concrete with improved physical and mechanical characteristics [11]. Multi-component modifiers on the chemical–mineral basis, the use of which allows to obtain a dense durable structure, finding practical application in construction [12]. In order to obtain self-compacting concrete mixtures with the optimal performance and to improve the operational properties of hardened concrete, the use of chemical and mineral modifiers is rational and promising [4]. Thus, improving the quality of self-compacting concrete is a problematic issue and requires its development taking into account the proposed technological method, which provides for the use of cement in its composition together with a complex modifier (hyperplasticizer + polymer + microsilica). It is further proposed to consider separately each component of the system and its role in the creation of self-compacting concrete with specified properties of strength, water resistance, and corrosion resistance [11].

After analyzing the requirements of construction companies, we proposed the idea of combining the useful properties of two types of concrete: SCC and fiber concrete—a composite material consisting of a cement matrix with a uniform distribution of oriented or chaotically arranged discrete fibers (fibers) of different origin in order to obtain a matrix that is as dense and resistant to hydrophysical effects as possible. Fiber concretes have enhanced deformation properties, primarily flexural tensile strength, low shrinkage deformations, and high abrasion resistance [8]. Having reviewed the scientific literature on hydrophysical indicators and the corrosion resistance of concrete, having become acquainted with the works of scientists in the field of building materials science on the investigation of the impact of various types of corrosion on the concrete matrix, and focusing on the peculiarities of the soils of Southern and Central Kazakhstan, we came to the conclusion that, in this work, it is necessary to investigate the indicators of corrosion resistance of modified SCC with the use of fiber reinforcement [7,13,14].

The study of the hydrophysical characteristics of concrete products and structures is a multifactorial scientific process consisting of the study of several physical and technical properties, since it is important not only to ensure the specified properties of concrete at the stage of manufacture but also to maintain them during the entire period of operation of the structure. In order to conduct further experiments, a number of research objectives were formed:

- investigation of the influence of the self-compacting concrete composition parameters and the amount of fiber reinforcement on the frost resistance of SCC and determination of the quantitative dependencies reflecting this most important performance indicator of SCC from the point of view of practical application [15];
- investigation of the influence of the self-compacting concrete composition parameters and the amount of fiber reinforcement on water absorption, water resistance, and porosity of SCC and determination of the quantitative dependencies reflecting this most important performance indicator of SCC from the point of view of practical application [16,17];
- determination of the corrosion resistance of SCC in conditions of aggressive impact of saline soils of the southern regions of the Republic of Kazakhstan [18].

The purpose of the work described in this article is to study the improvement of the operational properties and reliability of building products from self-compacting concrete, operated under conditions of repeated freezing–thawing and an aggressive environment of soils in Southern and Central Kazakhstan.

## 2. Materials and Methods

This work uses theoretical and applied research methods. The theoretical study was based on the fundamental laws of concrete science in order to study in detail and compare the corrosion resistance of self-compacting concretes of different compositions. The applied research was aimed at experimental confirmation of the theoretical hypothesis according to the current standards.

All the components of self-compacting concrete are extracted and produced in the territory of the Republic of Kazakhstan (Almaty region, Almaty city, Kazakhstan). All studies and tests were carried out in accordance with the regulatory documentation in force in the territory of the Republic of Kazakhstan.

### 2.1. Characterization of Concrete Mix Components

Cement CEM I 32.5H produced by “Standart Cement” LLP (Shymkent City, Kazakhstan) was accepted as a binder for the investigated concrete mixtures according to [19]. To confirm the compliance of the selected binder with the standards and requirements [20], a number of tests were carried out. The methods specified in these standards allow determining the following parameters of the binder:

*Fineness of grind:* Cement fineness is defined as the residue on the sieve, with mesh No. 008 as a percentage of the original weight of the sieved sample (to the nearest 0.1%). Cement is considered to meet the requirements of the normative documentation if at least 85% of the mass of the test sample has passed through the sieve [21]. The tested binder showed a grinding fineness of 92.8%.

*Normal density and setting time of cement batter:* The tested binder showed a normal density of 26.7% in the tests. The beginning of setting occurred after 2 h and 4 min, and the end of setting occurred after 4 h and 13 min from the moment of mixing with water. These values are within the normalized range.

*Compressive and flexural strength (at the age of 28 days):* When determining the strength characteristics of the studied binder, we showed the results at the age of 28 days: bending—5.3 MPa; compression—41.1 MPa. These indicators are included in the normative area.

For the tests, we used sand from the manufacturer “Giyada” LLP (Almaty Region, Almaty city, Kazakhstan), which complies with the normative document [22]. According to this standard, as fine aggregate for heavy concrete, under the definition of which will fall under the SCC, can be used sands with a maximum amount of dusty and clayey inclusions for the groups of increased coarseness, coarse, and medium in the amount of 3%. However, in order to obtain satisfactory characteristics of the concrete mix of the SCC, it is necessary to use sand, the amount of dust-like inclusions in which does not exceed 1.5%. The test for determining the amount of dusty and clayey inclusions of the considered sand was carried out by the method of scouring according to [21]. According to the test results, the content of dusty and clayey inclusions in the tested sand was 1.07%. The coarseness modulus of the tested sand was also determined, which was 2.6. These indicators are acceptable for the use of the investigated aggregate in SCC.

Crushed stone of fractions 5–10 mm and 10–20 mm produced by Novtehstroy LLP (Almaty Region, Almaty city, Kazakhstan) with known physical and technical characteristics was used as a coarse aggregate [23]. Grain composition of the aggregate meets the requirements of the normative document [24], which defines the basic requirements for crushed stone from dense rocks used as aggregate for heavy concrete, including SCC.

Characteristics of granite crushed stone: density—2850 kg/m^3^; water absorption—0.1%; crushed stone strength grade—12,000 kg·f/cm^2^; frost resistance—F 400; porosity—0.6%; dusty and clayey particles content—up to 1%; content of lamellar grains—11%; chemical composition: SiO_2_—49–64%; Al_2_O_3_—16–19%; CaO—3–10%; MgO—2.2–7.1%; Fe_2_O_3_—6.8–12.3%; SO_3_—0.4–0.9%.

Chemical additive based on polycarboxylate esters of the 2nd generation AR Premium produced by “Arirang Group” LLP (Astana, Kazakhstan) was used as the hyperplasticizer [25], with the following characteristics (Table 1):

As a water-soluble polymer additive, we used “polyvinylpyrrolidone 40.0”, a product of “Laborfarma” LLP (Almaty, Kazakhstan) with a molecular weight of 1,200,000–2,500,000 g/mol and viscosity from 3000 to 6000 MPa s at 25 °C, meeting the requirements [26].

By its nature, it is a synthetic polymer, not naturally occurring. Well miscible with alcohols, water-containing solutions, and chloroform. Soluble in water and polar solutions. Almost incompatible with ethers. Molecular formula: C_6_H_9_NO; molar mass: 2500—2,500,000 g/mol; density: 1200 kg/m³; melting point: 150–180 °C.

As a reactive pozzolanic additive was used microsilica MKU-95, produced by Tau-Ken Temir LLP (Karaganda, Kazakhstan), corresponding to [27,28]. Microsilica is formed by the reduction of high-purity quartz with coal in arc furnaces during the manufacture of silicon and ferrosilicon and consists of very fine spherical particles containing amorphous or glassy silicon dioxide (SiO_2_) in an amount of at least 85% of the additive weight. The composition and characteristics are summarized in Table 2.

For volumetric reinforcement in the research, chopped basalt fiber was used, manufactured in accordance with [29] and produced by LLP “Priority” Astana (Kazakhstan). The appearance and physical and mechanical characteristics of these fibers are given in Table 3.

### 2.2. Investigated Compositions of Modified Self-Compacting Concrete with Regard to the Consumption and Selection of Raw Materials

The task of increasing the strength and performance of the studied compositions of self-compacting concrete was solved by compacting its structure using a binder together with a complex modifier (hyperplasticizer AR Premium + polyvinylpyrrolidone 40.0 + microsilica MKU-95). To improve corrosion resistance, an active mineral admixture in the form of reactive microsilica was added to the concrete mix, and a basalt fiber was added to level the effect of bending moments.

In this test, the composition of self-compacting concrete of class C30/35 without the use of modifiers and fiber reinforcement [4] was taken as the control.

Tests to determine the required amount of each of the modifiers were carried out stepwise, starting from the control composition; with each subsequent test, hyperplasticizer AR Premium was introduced into the mixture at a rate of 1% of the binder per 1 m^3^, added to each subsequent test by 0.1% up to a rate of 2% per 1 m^3^; then, the tests were repeated with the introduction of the polymer additive. Polyvinylpyrrolidone 40.0 at a rate from 0.1% of the binder per 1 m^3^ was added to each subsequent test by 0.1% up to a rate of 1% per 1 m^3^ and, further, with the introduction of microsilica MKU-95 at a rate from 5% of the binder per 1 m^3^, added to each subsequent test by 1% up to a rate of 15% per 1 m^3^. The maximum amount of modifiers in the mixture was fixed based on previously published sources and on economic assumptions [30]. The optimum content of the complex admixture (1.5% AR Premium + 0.3% polyvinylpyrrolidone 40.0 + 15% MKU-95) was determined by the strength testing of 100 × 100 × 100 mm cube specimens tested at the age of 28 days of normal curing for each concrete composition. The compositions presented in Table 4 were used in further studies of physical–mechanical, hydrophysical characteristics, and corrosion resistance.

### 2.3. Concrete Compressive Strength

The compressive strength of concrete determines the resistance of the material to an applied mechanical compressive load. To determine the compressive strength of the investigated concrete compositions, three cube specimens of each composition with the dimensions of the working section 100 × 100 × 100 mm were prepared from the concrete mixture with the same water–cement ratio (W/C) and tested at the age of 28 days of normal curing according to the method by [31].

### 2.4. Determination of the Frost Resistance of Concrete

The method of repeated freezing and thawing in a water-saturated state was used for these tests. The test methodology and processing of the results were carried out in accordance with [15]. Water was used as the saturation medium. Air medium with a freezing temperature of minus 18 ± 2 °C was used and water with a temperature of 20 ± 2 °C as the thawing medium. Determination of the concrete frost resistance mark was carried out on specimens with ribs 100 × 100 × 100 mm at the age of 28 days, and the number of control specimens (6 pcs and main specimens—12 pcs for each composition) was established. The main specimens were saturated with water by immersion for 24 h to 1/3 of the height of the cube specimens before freezing and the control specimens before strength testing. The next step required us to increase the level to 2/3 of the height and continue the soaking for 24 h; after which, we increased the water level so that the distance from the top edge of the samples to the liquid level was more than 20 mm and continued the soaking for the next 48 h. For this study, the testing regime was strictly observed—the time of freezing the samples not less than 2.5 h and thawing for 2 ± 0.5 h; in a case of chipping, cracking, and flaking of ribs in the process of testing the sample, the study was stopped.

### 2.5. Determination of Water Resistance and Water Absorption of Concrete

The wet spot method was used to determine the water resistance. In accordance with the requirements of [16], we prepared cylinder samples with a diameter of 150 mm and height of 150 mm, because the largest grain size of the aggregate was 20 mm. For each investigated composition, 6 specimens were prepared, which were stored in a chamber of normal hardening with a set temperature of 20 ± 2 °C, relative humidity of 95 ± 5%, and preliminary curing for 1 day in the laboratory before testing. The process of testing, in the form of increasing water pressure, was carried out by loading in steps of 0.2 MPa for 1–5 min and the duration of loading in each step equal to 12 h. Water resistance of each cylinder specimen was assessed by fixing the maximum value of water pressure at which no water seepage through the specimen body in the form of wet spots or signs of water filtration in the form of drops was observed on the end surface of the specimen opposite to its surface through which water was pressurized. To determine the water tightness of a series of concrete specimens, the maximum water pressure at which no water filtration was observed on at least four specimens out of six was evaluated.

The water absorption study was carried out according to the requirements of [17]. For this purpose, we used cube samples with dimensions 100 × 100 × 100 mm, based on the largest grain size of aggregates of 20 mm, 3 samples of each composition, which were placed in a container with water at the temperature 20 ± 2 °C, so that the water level was higher by 50 mm above the top edge of the sample. Every 24 h of water absorption, the samples were weighed until two consecutive weighing differed by no more than 0.1%. The results were processed by determining the water absorption of a single concrete sample by weight (*W_m_*) in percent and an error of no more than 0.1% according to Formula (1):

(1) $$W_{m}=\frac{m_{c}-m_{v}}{m_{c}}\times 100$$

where *W_m_*—water absorption of individual concrete specimen by mass, %; *m_c_*—mass of dried sample, g; *m_v_*—mass of the water-saturated sample, g.

To determine the water absorption of a series of samples of each concrete composition, the arithmetic mean of the obtained results of an individual sample was calculated.

Determination of the porosity of the concrete cube specimens with rib size 70 × 70 × 70 mm series of two specimens for each composition was carried out by water absorption kinetics, a discrete weighing method.

### 2.6. Determination of the Corrosion Resistance of the Concrete Specimens

The essence of the test method in accordance with [18] is a comparative analysis of the obtained results of the examination of test samples placed in a non-aggressive environment with the values of the indicators of samples of the same composition placed in an aggressive environment. For each formulation, 3 control samples and 3 basic samples were prepared for each aggressive medium investigated. The dimensions are based on the largest aggregate size and are set as recommended, with rib dimensions of 100 × 100 × 100 mm. Studies were carried out on cube specimens aged 28 days of normal curing in aqueous solutions of 5% sodium sulfate (Na_2_SO_4_), 3% sodium chloride (NaCl), distilled water, and 0.01 M hydrochloric acid (HCl) solution. Bottled drinking water was used as a non-aggressive medium [32]. The samples were dried and weighed before starting the studies. In the next step, three control specimens were tested for flexural tensile strength and compressive strength. The prepared three specimen prisms of each composition were placed in the above aggressive media, ensuring its uniform access to the specimen from all sides, with a study duration of 6 months.

Processing of the test results was carried out by comparing the properties of the specimens in an aggressive environment and specimens cured in drinking water. Firstly, the mass loss of samples (Δ*m*) % was estimated by Formula (2):

(2) $$\Delta m=\frac{m_{1}-m_{2}}{m_{1}}\times 100\%$$

where Δ*m*—mass loss of samples, %; *m*_1_—mass of the specimen before the test, g; *m*_2_—mass of the specimen after the test, g.

At the next stage, we needed to establish the change in tensile strength in bending (ΔR_tb,%) and compressive strength (ΔR, %) by Formulas (3) and (4):

(3) $$\Delta R=\frac{R_{1}-R_{2}}{R_{1}}\times 100\%$$

where ΔR—change in compressive strength, %; R_1_—compressive strength of the control specimens (before testing), MPa; R_2_—compressive strength of the main specimens (at the end of the test), MPa.

(4) $$\Delta R_{\mathrm{tb}}=\frac{R_{1\mathrm{tb}}-R_{2\mathrm{tb}}}{R_{1\mathrm{tb}}}\times 100\%$$

where ΔR_tb_—change in tensile strength in bending, %; R_(1 tb)_—tensile strength in bending of the control specimens (before testing), MPa; R_(2 tb)_—tensile strength in bending of the main specimens (at the end of the test), MPa.

## 3. Results

In the operation of products and structures made of self-compacting concrete, practical experience has shown that these structures are not durable and reliable enough. There are numerous cases of premature failure of some elements due to alternate freezing and thawing and corrosion of concrete, resulting in the need for costly repairs. It is necessary to take into account at once the factor of influence of an aggressive environment and the operating conditions of the structure, to choose the right raw materials [33].

### 3.1. Compressive Strength

**Figure 1:**
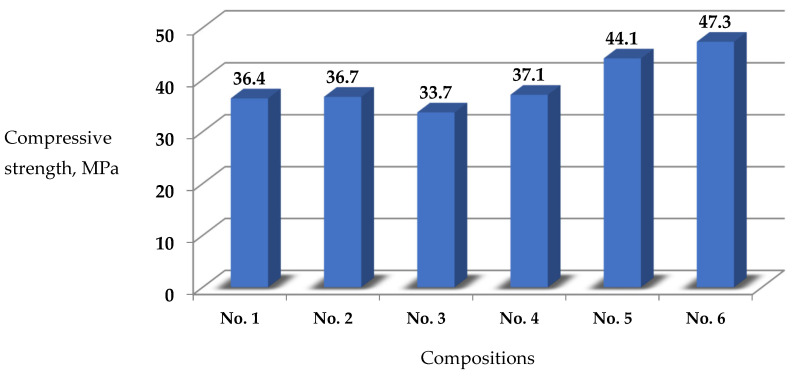
Compressive strength test results of self-compacting modified concrete (average values). Compositions No. 1—Control Zavodskoy (Class C30/35); No. 2—1.5% AR Premium; No. 3—0.3% polyvinylpyrrolidone 40.0; No. 4—1.5% AR Premium + 0.3% polyvinylpyrrolidone 40.0; No. 5—1.5% AR Premium + 0.3% polyvinylpyrrolidone 40.0 + 15% MKU-95; No. 6—(1.5% AR Premium + 0.3% polyvinylpyrrolidone 40.0 + 15% MKU-95) + 0.7% basalt fiber.

The compression test results of the modified self-compacting concrete specimens are presented in Table 5 (Figure 1).

The obtained data of the presented studies (Table 5) allow us to establish the following scientific observations:

- an increase in the compressive strength of composition 5 (1.5% AR Premium + 0.3% polyvinylpyrrolidone 40.0 + 15% MKU-95) by 21% relative to the control composition 1 and 20% relative to composition 2 (PC + 1.5% AR Premium) was established, which is in absolute terms by 7.7 and 7.4 MPa, respectively. When a complex modifier (1.5% AR Premium + 0.3% polyvinylpyrrolidone 40.0 + 15% MKU-95) is introduced into the concrete mixture, the processes of hydrolysis and hydration of cement particles are more intensive with the formation of additional crystallization centers, which is confirmed by earlier microstructural analyses of cement stone [34].
- found that, by adding basalt micro-reinforcing fiber with a fiber concentration of 0.7% of the binder weight to the proposed composition 6, a slight increase in strength by 7.2% in relation to composition 5 (without fiber) is observed. It should also be noted that, when the fiber content increases above 1% of the binder, the clumping of fibers is observed, which negatively affects the strength of concrete. The obtained results are in agreement with the work [30].

### 3.2. Determination of Frost Resistance of Modified Self-Compacting Concrete

In the next stage of research, frost resistance was determined, which depends on the structure and nature of the pores in concrete. The relationship between porosity and frost resistance is a complex and interesting process, as evidenced by the use of an air-entraining admixture in concrete by some researchers to create closed pores to increase its workability and frost resistance, but the amount of air-entraining admixture must be appropriate to maintain the strength of concrete. The presence of open pores available for water penetration has a bad effect on frost resistance and durability [35].

The frost resistance of concrete is directly dependent on its structure, as it determines the volume and distribution of ice formed in the concrete body at sub-zero temperatures and, consequently, the value of the resulting stresses and the intensity of the increasing weakening process of the structure. Concrete micropores of ≈10^−5^ cm usually contain bound water, which does not convert to ice even at very low temperatures (down to −70 °C). Therefore, micropores have no noticeable effect on the frost resistance of concrete, and it depends on the volume of macropores in concrete [36].

For frost resistance tests, the first basic method according to the requirements of [15] was used for repeated freezing and thawing in a water-saturated state. Determination of the frost resistance grade of concrete was carried out on specimens with 100 × 100 × 100 mm ribs at the age of 28 days. The results of the tests at cyclic alternate freezing and thawing are shown in Table 6.

The results of the tests at cyclic alternate freezing and thawing of the concrete cube samples of different compositions presented in Table 6 showed:

- a maximum weight loss up to 4.33% in the control composition 1 after 400 test cycles of alternate freezing and thawing, which exceeds the established indicators of the requirement [15] (weight loss not more than 2%).
- The compositions containing the complex modifier (1.5% AR Premium + 0.3% polyvinylpyrrolidone 40.0 + 15% MKU-95) showed high frost resistance characteristics. After 600 cycles of tests, the mass loss in compositions 5 and 6 was 1.9% and 1.6%, which confirms the sufficient frost resistance reserve of the proposed compositions of modified self-compacting concrete.

### 3.3. Determination of Water Resistance and Water Absorption of Concrete

When building structures from SCC in the conditions of an aggressive environment and contact with water, the concrete should be dense and have good hydrophysical properties: low indicators of water absorption, capillary suction, water resistance, and high indicators of frost and corrosion resistance. In works [37,38], the improvement of the hydrophysical properties of concrete with the use of modifiers, especially, which include ingredients of hydrophobic-plasticizing action by 20% and more, is noted. We have carried out standard tests according to the methodology described in Section 2.5 of the proposed modified self-compacting concrete compositions. The results of the water absorption and water resistance tests are presented in Table 7.

**Figure 2:**
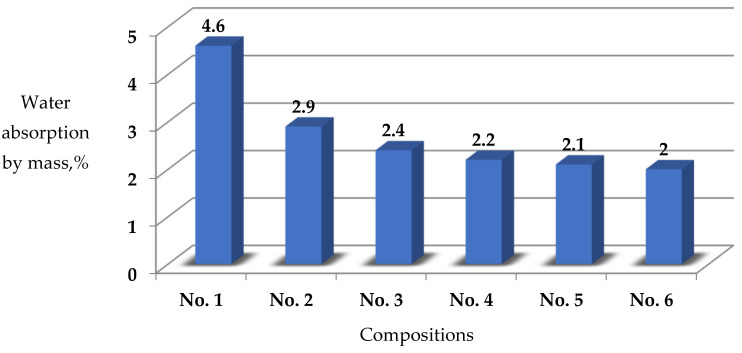
Water absorption test results. compositions No. 1—control Zavodskoy (Class C30/35); No. 2—1.5% AR Premium; No. 3—0.3% polyvinylpyrrolidone 40.0; No. 4—1.5% AR Premium + 0.3% polyvinylpyrrolidone 40.0; No. 5—1.5% AR Premium + 0.3% polyvinylpyrrolidone 40.0 + 15% MKU-95; No. 6—(1.5% AR Premium + 0.3% polyvinylpyrrolidone 40.0 + 15% MKU-95) + 0.7% basalt fiber.

Analysis of the obtained data in Table 7 (Figure 2) shows that the concrete sample with the complex modifier together with basalt fiber (composition 6) reduced the water absorption index of the concrete by 56.5%, composition 2 by 37%, composition 3 by 47.8%, composition 4 by 52.2%, and composition 5 by 54.3% compared to the control (composition 1).

The water resistance of the modified SCC in composition 5) increased by 0.8 MPa or four grades (marks) in comparison to the control (composition 1). At the same time, the presence of basalt fiber in composition 6 did not affect its water resistance in comparison to composition 5.

### 3.4. Determination of the Corrosion Resistance

Solutions of salts of low concentration are not aggressive to SCC at the constant immersion of structures in them, but at alternate saturation and drying or at the capillary absorption of such a solution, the concentration of the solution in drying concrete and their crystallization in concrete pores (salt form of concrete corrosion) are possible.

The saturation of SCC with salts containing chloridiones (NaCl chlorides) may cause the corrosion of steel reinforcement of the structure, i.e., such salts are aggressive towards reinforced concrete. Based on the analysis of the causes of corrosion in concrete and the assessment of the degree of aggressiveness of the media in relation to reinforced concrete structures, it follows that corrosion resistance depends both on the conditions of interaction between concrete and the external environment and on the composition of the influencing aggressive solution [39].

In this work, we studied the effect on self-compacting concrete of different liquid aggressive media simulating all three types of corrosion according to [14]. The obtained test results are given in Table 8.

The results of the study of resistance to aggressive influences of SCC samples by changes in the average mass, tensile strength in bending, and compression (Table 8) showed that the proposed composition 6 has a high resistance to corrosion. When the specimens were kept in 3% NaCl solution, the mass loss Δmcr was 0.091%, compressive strength ΔR_av_. Was 0.144%, and bending strength ΔR_tb was 0.131%, respectively.

During corrosion testing in 0.01 mol/L HCl hydrochloric acid solution, the following changes were observed in composition 6: decrease in the reduction of the average mass loss Δm_av._ by 46.4%, compressive strength ΔR_av._ by 56%, and flexural tensile strength ΔR_tb by 65.6% compared to the control (composition 1), respectively.

When tested by leaching corrosion in distilled water, the following changes were observed in composition 6: decrease in the reduction of the average weight loss Δm_av._ by 21.5%, compressive strength ΔR_av._ by 48.8%, and flexural tensile strength ΔR_tb by 49.6% compared to the control (composition 1), respectively.

**Figure 3:**
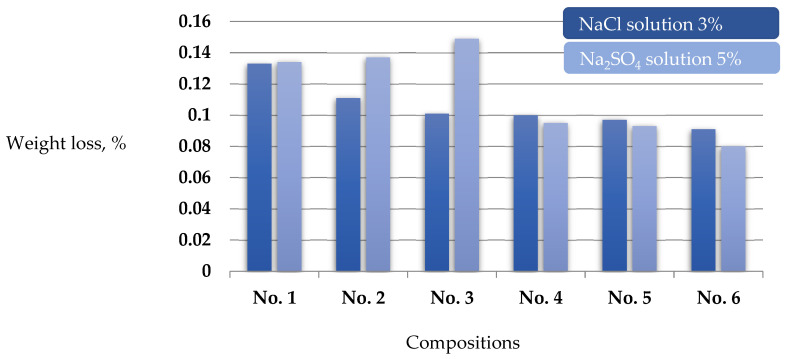
Resistance to the aggressive environment of SCC samples by the change in average mass. Compositions No. 1—control Zavodskoy (Class C30/35); No. 2—1.5% AR Premium; No. 3—0.3% polyvinylpyrrolidone 40.0; No. 4—1.5% AR Premium + 0.3% polyvinylpyrrolidone 40.0; No. 5—1.5% AR Premium + 0.3% polyvinylpyrrolidone 40.0 + 15% MKU-95; No. 6—(1.5% AR Premium + 0.3% polyvinylpyrrolidone 40.0 + 15% MKU-95) + 0.7% basalt fiber.

When tested (Figure 3) for the aggressive effect of sulfate in 5% solution (Na_2_SO_4_), the increased performance was inversely shown by the specimens (composition 6). Reductions in the mass loss Δm_av._ by 40.3%, compressive strength ΔR_av._ by 38.4%, and flexural tensile strength ΔR_tb by 46.9% relative to the control (composition 1) were observed, respectively. The obtained test results are in agreement with the results of [33,40].

## 4. Discussion

The results of compressive strength studies of SCC show a significant improvement in the strength properties in the modified formulations. For example, composition 5 containing 1.5% AR Premium, 0.3% polyvinylpyrrolidone 40.0, and 15% MKU-95 shows an increase in strength of 21% over control composition 1 and 20% over composition 2. This confirms the effectiveness of using these additives to improve the strength properties of concrete.

It is interesting to note that the basalt micro-reinforcing fiber in composition 6 has only a minor effect on increasing the compressive strength of concrete, increasing it up to 7% compared to composition 5 without fiber. This indicates that fiber only plays a positive role in improving the concrete flexural strength and deformation under bending loads.

The use of a complex modifier including 1.5% AR Premium, 0.3% polyvinylpyrrolidone 40.0, 15% MKU-95, and 15% microsilica leads to a number of positive effects. This includes a reduction in the water–cement ratio, an increase in strength according to the basic law of concrete strength, and an increase in density, which favors hydrophysical properties and corrosion resistance. The water-soluble polymer additive “polyvinylpyrrolidone 40.0” modifies the pore space of cement stone, thereby increasing its impermeability and frost and corrosion resistance [41]. Microsilica increases the density and strength of cement stone and concrete based on it, as well as resistance to type I corrosion (leaching) due to the binding of Portlandite Ca(OH)_2_ into water-soluble low-base calcium hydrosilicates [42]. Basalt fiber increases the crack resistance by its micro-reinforcement, the result of which is the leveling and reduction of stress concentration in the concrete structure, particularly in the macro-defect zone [43].

The conducted hydrophysical tests show a decrease in water absorption of the complex modifier-modified SCC and an increase in its water resistance.

The received positive results of the tests confirm the fact of an increase of pozzolanic activity to free Ca(OH)_2_ of microsilica MKU-95 contained in composition 6 together with basalt fiber possessing chemical resistance that speaks about the conformity of the results of the research with the theoretical assumptions about the occurrence of additional centers of crystallization and decrease of pore space in a body of concrete by reactions of active pozzolanic additives (active microsilica SiO_2_); there is a process of binding of Ca(OH)_2_ by active mineral fiber—SiO_2_—into a poorly soluble compound—calcium hydrosilicate—according to the equation Ca(OH)_2_ + SiO_2_ + mH_2_O = CaO·SiO_2_·nH_2_O [44].

When studying the soils encountered in the south of Kazakhstan and their classifications, it is necessary to note those that has been a high degree of aggressive impact on concrete structures. For example, saline soils of brown soils have pH 2.8–3.5; gray-brown soils of the steppe zone are less aggressive, and their pH is in the range of 4.5–5.5. Loamy soils are composed of sodium bicarbonate (NaHCO_3_) and sodium carbonate (Na_2_CO_3_) and are classified as alkaline soils with an elevated pH greater than 8.5. Corrosion processes associated with the leaching of calcium hydroxide Ca(OH)_2_ occurring in concrete under the action of soft water belong to the first group of corrosion. Corrosion of the second group includes corrosion processes associated with the process of the interaction of cement stone and calcium hydroxide with an aggressive medium, resulting in the formation of easily leachable and easily soluble calcareous compounds or an increase in volume, which is accompanied by a reaction: Ca(OH)_2_ + CO_2_ = CaCO_3_ + H_2_O. Calcium carbonate (CaCO_3_) is insoluble in water. Over time, it deposits in the pores of the cement stone, increasing the volume of the concrete and, as a result, increasing cracking and deterioration. However, calcium carbonate is able to further interact with the carbon dioxide in the water to form a soluble acidic salt, causing the carbon dioxide corrosion of concrete: CaCO_3_ + H_2_O + CO_2_ → Ca(HCO_3_)_2_.

Sulfate corrosion is one of the most common types of chemical deterioration of cement-based building materials. In contact with concrete, sulfates actively interact with calcium hydroxide and aluminate constituents of cement stone. As a result of the reaction of sulfates with calcium hydroxide, gypsum is formed, the accumulation and increase of which in the pore space of concrete leads to its destruction. At a high concentration of sulfates in the liquid phase, an excess concentration of SO_4_^−2^ anions appears in the solution, which react with calcium cations: Ca^2+^ + SO_4_^−2^ → CaSO_4_ ∙ 2H_2_O; the resulting gypsum is saturated with water and increases in volume during crystallization, which leads to the destruction of the cement stone.

In order to obtain complete data on the corrosion resistance of modified self-compacting concrete, we conducted experiments to identify the resistance of concrete to three types of corrosion according to the classification of V.M. Moskvin [45]. The corrosion resistance studies showed that the complex modifier-modified SCC formulations have a high degree of resistance in aggressive environments and leaching corrosion. The use of modifiers allowed reducing the mass loss of the specimens during corrosion leaching up to 50%, loss of compressive strength ΔR_av._ up to 40%, and flexural tensile strength ΔR_tb up to 60% as compared to the control specimens without modifiers.

## 5. Conclusions

Modified by a complex modifier (hyperplasticizer + polymer + microsilica + basalt fiber), SCC compositions show high strength, frost resistance, and resistance in aggressive environments. The introduction of a certain amount of complex modifier increases the durability of the current SCC compositions, increasing water resistance by four grades up to W16, frost resistance up to F = 500, increasing the compressive strength by 20%, and reducing the mass loss of the samples during corrosion leaching up to 50%. This emphasizes the effectiveness of the proposed complex modifier in improving the properties of SCC, especially since the constituents of the complex modifier are inexpensive and readily available. Taking into account the results of the conducted work, it should be noted that complex modifiers for concrete would be further recommended for practical application in Kazakhstan.

## Figures and Tables

**Table 1:** Technical characteristics of the AR Premium hyperplasticizer.

Indicator	Indicator Value
Appearance	Homogeneous light yellow-colored liquid
Density at 25 °C, kg/m^3^	1030–1060
Hydrogen index, pH	3.7
Chloride content, not more (%)	0.1

**Table 2:** Chemical composition of microsilica.

**Condensed microsilica ST ORG 46976-1901-21-002-2014 (ST ORG—Standard of organization)**	**Mark**	**Batch No.**	**Mass Ratio** **, %**						
	MKU	27	SiO_2_	Fe_2_O_3_	Al_2_O_3_	CaO	pH	q, g/cm^3^	Other impurities
			96.91	0.07	0.24	0.46	7.89	0.44	2.77

**Table 3:** Physical and mechanical characteristics of chopped basalt fiber.

Indicator	Characteristics
	Basalt Fiber
Melting point, °C	1450
Length of section, mm	12.67
Resistance to alkalis and corrosion	High
Elementary fiber diameter, mcm	16.19
Elongation at break, %	1.4–3.6
Tensile strength, R, MPa∙10^3^	2.8–3.4
Density, g/cm^3^	2.63
Modulus of elasticity F_e_, MPa∙10^3^	100–130

**Table 4:** Investigated compositions of modified self-compacting concrete.

Materials	Composition and Consumption per 1 m^3^ of Concrete Mix, kg/m^3^					
	Composition 1 (Control.)	Composition 2	Composition 3	Composition 4	Composition 5	Composition 6
Cement I 32.5H	460	460	460	460	400	400
Microsilica MKU-95 (15%)	-	-	-	-	60	60
Water	210	210	210	210	210	210
Granite crushed stone (5–10) mm	730	730	730	730	730	730
Granite crushed stone (10–20) mm	170	170	170	170	170	170
Sand	820	820	820	820	820	820
Hyperplasticizer “AR Premium” (1.5%)	-	6.9	-	6.9	6.9	6.9
Polyvinylpyrrolidone 40.0 (0.3%)	-	-	1.38	1.38	1.38	1.38
Basalt fiber (BF) (0.7%)	-	-	-	-	-	3.22
Flowability (mm)	560	720	570	700	690	680

**Table 5:** Compressive strength test results of self-compacting modified concrete.

Sample Marking	No. of Sample	Density, kg/m^3^	Compressive Strength, MPa	
			R_compr._	R_compr._, Average
Composition 1 Control Zavodskoy (Class C30/35)	1	2377	35.5	36.4
	2	2379	36.7	
	3	2395	37.1	
Composition 2 1.5% AR Premium	1	2368	36.2	36.7
	2	2374	36.6	
	3	2382	37.2	
Composition 3 0.3% Polyvinylpyrrolidone 40.0	1	2394	33.4	33.7
	2	2395	34.1	
	3	2399	33.6	
Composition 4 1.5% AR Premium + 0.3% Polyvinylpyrrolidone 40.0	1	2401	37.5	37.1
	2	2397	37.0	
	3	2393	36.8	
Composition 5 (1.5% AR Premium + 0.3% Polyvinylpyrrolidone 40.0 + 15% MKU-95)	1	2400	44.0	44.1
	2	2401	44.1	
	3	2402	44.2	
Composition 6 (1.5% AR Premium + 0.3% Polyvinylpyrrolidone 40.0 + 15% MKU-95) + 0.7% basalt fiber	1	2401	47.9	47.3
	2	2400	46.9	
	3	2396	47.1	

**Table 6:** Results of concrete testing under cyclic alternating freezing and thawing.

Sample Marking	Sample Weight Loss, %, after Cycles						K_frost._ after Cycles					
	200	300	350	400	500	600	200	300	350	400	500	600
Composition 1 Control Zavodskoy (Class C30/35)	1.19	2.17	3.16	4.33	-	-	0.95	0.9	0.77	0.68	-	-
Composition 2 1.5% AR Premium	0.46	1.13	1.19	2.1	3.7	5.31	1.01	0.95	0.93	0.91	0.83	0.81
Composition 3 0.3% Polyvinylpyrrolidone 40.0	0.33	1.08	1.55	2.01	3.3	4.84	1.03	0.99	0.96	0.95	0.87	0.84
Composition 4 1.5% AR Premium + 0.3% Polyvinylpyrrolidone 40.0	0.24	0.99	1.61	1.92	2.8	3.9	1.04	1.0	0.98	0.97	0.95	0.89
Composition 5 1.5% AR Premium + 0.3% Polyvinylpyrrolidone 40.0 + 15% MKU-95	0.11	0.95	1.1	1.5	1.7	1.9	1.06	1.03	0.99	0.97	0.96	0.92
Composition 6 (1.5% AR Premium + 0.3% Polyvinylpyrrolidone 40.0 + 15% MKU-95) + 0.7% basalt fiber	0.09	0.29	0.27	0.42	1.4	1.6	1.11	1.09	1.03	1.0	0.98	0.94

Note: K_frost._—ratio of the strength index of the sample after the frost resistance test to the strength of the material sample in the water-saturated state before the frost resistance determination. In frost resistance testing, the frost resistance coefficient K_frost._ = R_frost._/R_c_ is used to determine the actual change in strength after a given number of cycles; R_frost._—concrete strength after the accepted number of test cycles; R_c_—strength of control samples in a water-saturated state. The frost resistance grade of concrete is considered to be secured after the required number of cycles if K_frost._ > 0.95.

**Table 7:** Results of the water absorption and water resistance tests.

Sample Marking	Water Absorption by Weight, %	Water Resistance, MPa	Concrete Waterproof Mark
Composition 1 Control Zavodskoy (Class C30/35)	4.6	0.6	W6
Composition 2 1.5% AR Premium	2.9	0.8	W8
Composition 3 0.3% Polyvinylpyrrolidone 40.0	2.4	1.0	W10
Composition 4 1.5% AR Premium + 0.3% Polyvinylpyrrolidone 40.0	2.2	1.2	W12
Composition 5 (1.5% AR Premium + 0.3% Polyvinylpyrrolidone 40.0 + 15% MKU-95)	2.1	1.4	W14
Composition 6 (1.5% AR Premium + 0.3% Polyvinylpyrrolidone 40.0 + 15% MKU-95) + 0.7% basalt fiber	2.0	1.4	W14

**Table 8:** Resistance to corrosive environment of SCC samples by change of the average mass, compressive strength, and flexural tensile strength.

Sample Marking	Characteristics	Type and Concentration of Aggressive Medium			
		NaCl Solution 3%	Distilled Water	HCl 0.01 mol/L	Na_2_SO_4_ Solution 5%
Composition 1 Control Zavodskoy (Class C30/35)	Δm_av._, %	0.133	0.065	0.233	0.134
	ΔR_av._, %	0.242	0.121	0.539	0.211
	$\Delta R_{tb}$_av._, %	0.254	0.129	0.611	0.288
Composition 2 1.5% AR Premium	Δm_av._, %	0.111	0.055	0.185	0.137
	ΔR_av._, %	0.185	0.101	0.391	0.223
	$\Delta R_{tb}$_av._, %	0.18	0.113	0.416	0.274
Composition 3 0.3% Polyvinylpyrrolidone 40.0	Δm_av._, %	0.101	0.056	0.184	0.149
	ΔR_av._, %	0.173	0.095	0.375	0.195
	$\Delta R_{tb}$_av._, %	0.164	0.101	0.409	0.269
Composition 4 1.5% AR Premium + 0.3% Polyvinylpyrrolidone 40.0	Δm_av._, %	0.1	0.047	0.12	0.095
	ΔR_av._, %	0.16	0.082	0.29	0.141
	$\Delta R_{tb}$_av._, %	0.105	0.081	0.27	0.172
Composition 5 (1.5% AR Premium + 0.3% Polyvinylpyrrolidone 40.0 + 15% MKU-95)	Δm_av._, %	0.097	0.05	0.122	0.093
	ΔR_av._, %	0.156	0.074	0.241	0.145
	$\Delta R_{tb}$_av._, %	0.137	0.075	0.22	0.165
Composition 6 (1.5% AR Premium +0.3% Polyvinylpyrrolidone 40.0+15% MKU-95)+ 0.7% basalt fiber	Δm_av._, %	0.091	0.051	0.125	0.08
	ΔR_av._, %	0.144	0.062	0.237	0.13
	$\Delta R_{tb}$_av._, %	0.131	0.065	0.21	0.153

## Data Availability

Data are contained within the article.
